# Case Report: Synchronous SCLC and minimally invasive adenocarcinoma in the same lobe: NGS-confirmed cross-histological molecular heterogeneity in an ultra-rare case

**DOI:** 10.3389/fonc.2026.1844056

**Published:** 2026-08-27

**Authors:** Haoyuan Yin

**Affiliations:** Department of Thoracic Surgery, Yuxi People’s Hospital, The Sixth Affiliated Hospital of Kunming Medical University, Yuxi, Yunnan, China

**Keywords:** minimally invasive adenocarcinoma, molecular heterogeneity, next-generation sequencing, rapid tumor growth, small cell lung cancer, synchronous lung cancer

## Abstract

A 73-year-old male was diagnosed with synchronous multiple primary lung cancer (sMPLC) involving small cell lung cancer (SCLC) and minimally invasive adenocarcinoma in the right upper lobe—an ultra-rare entity accounting for only 2.0% of all lung cancers, with cross-histological (SCLC + adenocarcinoma) same-lobe presentation reported in fewer than 10 cases globally to date. Notably, the SCLC component exhibited striking aggressiveness and diagnostic mimicry: it initially presented as a 0.3cm tiny nodule with benign imaging features (clear borders, no lobulation), which was consistent with “low-risk nodule” criteria per clinical guidelines, yet rapidly progressed to 1.8cm×1.5cm×1cm within 1 year (a 31-fold volume increase). The patient underwent single-port video-assisted thoracoscopic right upper lobectomy combined with mediastinal lymph node dissection. Pathological examination confirmed: Nodule 1 (the progressed lesion) was peripheral SCLC with airway spread (Syn, CgA, CD56 positive); Nodule 2 (0.8cm×0.4cm×0.3cm) was peripheral minimally invasive adenocarcinoma with predominant lepidic growth pattern (TTF-1, Napsin A positive). No mediastinal lymph node metastasis was detected (Groups 2 + 4/7/10-14, 0/15). Next-generation sequencing (NGS) of mixed tissue from both lesions revealed an EGFR exon21 c.2573T>G mutation (p.L858R, 0.46%, OncoKB Level 1 sensitivity) and a TP53 exon8 c.820G>T mutation (p.V274F, 79.5%), with no RB1 mutation detected; although this is consistent with *de novo* SCLC, it is insufficient to exclude transformation given that 20–30% of confirmed T-SCLC cases lack RB1 mutations. Four cycles of postoperative etoposide plus cisplatin chemotherapy were well tolerated, with no severe adverse events. Eight-month follow-up showed no tumor progression; a slight elevation of progastrin-releasing peptide (ProGRP, 136.70ng/L) was considered a postoperative transient fluctuation. This case highlights the extreme rarity of same-lobe cross-histological synchronous SCLC and adenocarcinoma, as well as the “benign mimicry” and ultra-fast progression of small-volume SCLC—key pitfalls in preoperative diagnosis. Preoperative multimodal evaluation, postoperative immunohistochemistry, and NGS facilitate accurate diagnosis, while single-port thoracoscopy combined with platinum-based chemotherapy constitutes a safe and effective treatment strategy.

## 1 Introduction

Synchronous multiple primary lung cancer (sMPLC) is defined as multiple primary lung malignancies diagnosed within 6 months, in accordance with the Martini-Melamed criteria (1). Most sMPLC cases involve subtypes of non-small cell lung cancer (NSCLC), while cross-histological cases combining SCLC and adenocarcinoma in the same lobe account for less than 0.1% of reported cases (2). Such cases pose unique diagnostic and therapeutic challenges due to the distinct pathological and biological characteristics of the two tumor types. Current clinical gaps include unclear mechanisms underlying short-term lesion progression (<1 year), lack of standardized postoperative chemotherapy regimens, and unestablished consensus on the clinical value of molecular testing (3). This report presents a case of synchronous SCLC and minimally invasive adenocarcinoma in the right upper lobe, and discusses diagnostic and therapeutic strategies with evidence-based insights.

## 2 Case presentation

### 2.1 Baseline patient information

A 73-year-old male was incidentally found to have bilateral pulmonary nodules during a routine health examination. He had no respiratory symptoms such as cough, dyspnea, or chest pain at the time of presentation.

### 2.2 Preoperative examinations

#### 2.2.1 Imaging studies

**Figure 1:**
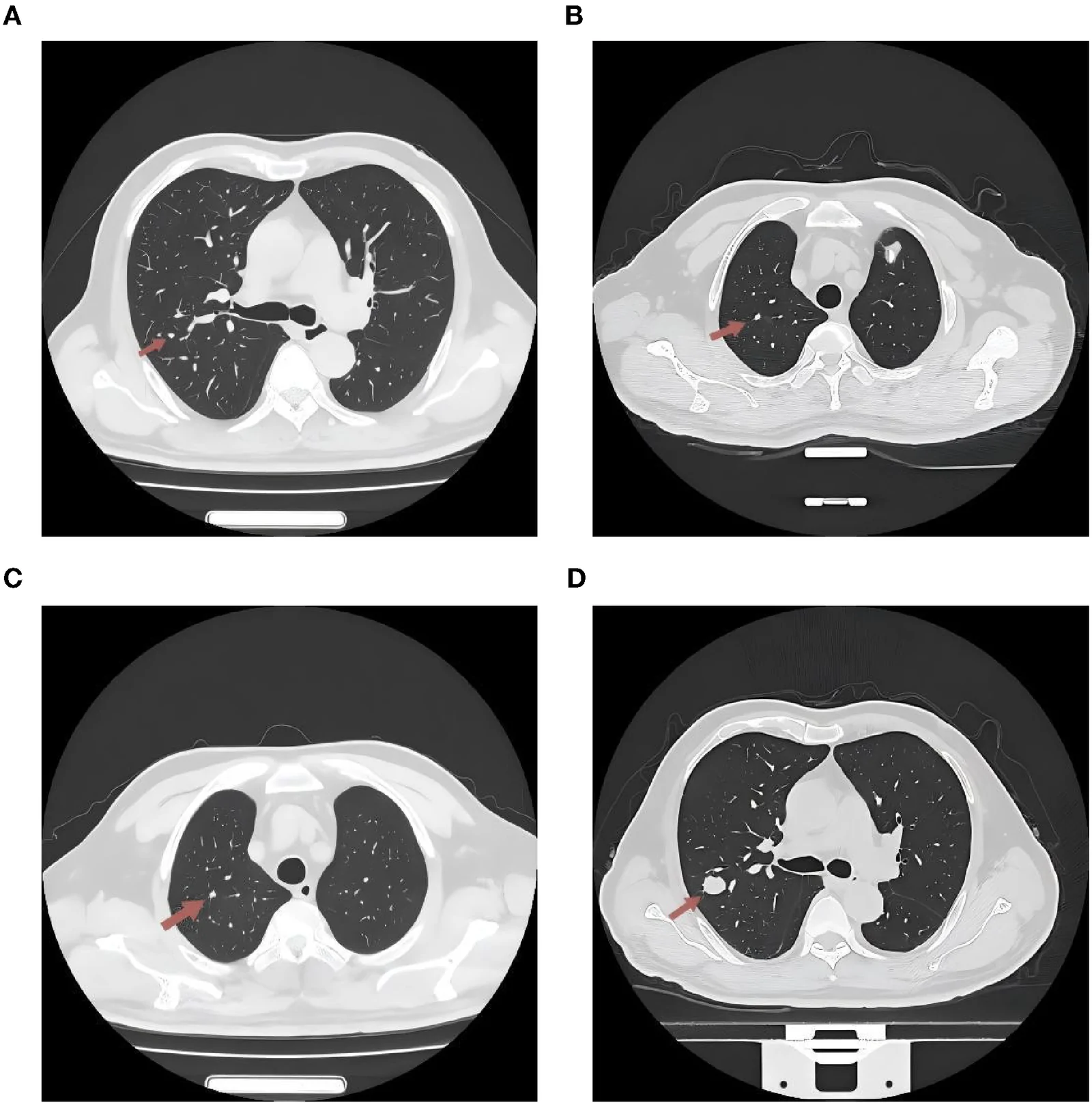
**(A)** January 2024 CT image showing a small solid nodule in the posterior segment of the right upper lobe (red arrow). **(B)** February 2025 CT image showing a stable nodule in the apical segment of the right upper lobe (red arrow). The faint opacity visible in the left upper lobe in this image is an artifact caused by respiratory motion, confirmed on adjacent CT slices which showed no corresponding parenchymal lesion. **(C)** January 2024 CT image showing the nodule in the apical segment of the right upper lobe (red arrow). **(D)** February 2025 CT image showing the enlarged lesion in the posterior segment of the right upper lobe, measuring 1.7 cm × 1.5 cm, with lobulation sign (red arrow). The posterior segment nodule was confirmed as SCLC (panels **A** and **D**); the apical segment nodule was confirmed as MIA (panels **C** and **B**).

- The patient underwent chest CT as part of a routine health examination due to his age and history of coronary heart disease. January 2024 Chest CT: Multiple solid nodules were detected in both lungs. CT imaging revealed a small 0.3 cm solid nodule in the apical segment of the right upper lung lobe (**Figure 1C**), as well as a 0.8 cm nodule in the posterior segment of the right upper lung lobe (**Figure 1A**).
- February 2025 Chest CT: The nodule in the posterior segment enlarged to 1.7cm×1.5cm, with lobulation and rough edges (**Figure 1B**), while the nodule in the apical segment remained unchanged in size (**Figure 1D**). A motion-related artifact was noted in the left upper lobe on this scan, confirmed as non-pathological on adjacent slices.

#### 2.2.2 Bronchoscopy and preoperative pathological examination

Brush cytology suggested “a small amount of atypical cells, favoring neuroendocrine tumor.” Intrapulmonary metastasis was excluded based on: (1) the distinct pathological types of the two nodules (SCLC vs. MIA); (2) the independent clonal origin suggested by contrasting NGS allele frequencies (EGFR 0.46% vs. TP53 79.5%); (3) absence of mediastinal lymph node metastasis; and (4) compliance with Martini-Melamed criteria for multiple primary lung cancers (1).

#### 2.2.3 Multidisciplinary team discussion

sMPLC was confirmed; the differentiation between multiple primary lung cancers and intrapulmonary metastasis was established based on the criteria described above.

### 2.3 Surgical procedure

- Surgery Date: February 28, 2025
- Operative Details: A 3cm incision was made at the 5th intercostal space on the right chest. Right upper lobectomy was performed, followed by systematic mediastinal lymph node dissection (Groups 2 + 4, 7, and 10-14). Operative time was 60 minutes; intraoperative blood loss was 50ml; postoperative 24-hour chest tube drainage volume was <100ml.
- Specimen Handling: Nodule 1 was marked with a single suture, and Nodule 2 was marked with double sutures. Both nodules were sent for pathological examination immediately after resection.

### 2.4 Pathological results

The clinicopathological characteristics of both nodules are summarized in **Table 1**.

#### 2.4.1 Gross specimen description

- Right upper lobe (measuring 17cm×10cm×2.5cm): Nodule 1 (single suture marker) was a gray-red solid tumor, 1.8cm×1.5cm×1cm in size, located 4.5cm from the staple line. Nodule 2 (double suture marker) was a gray-white lesion, 0.8cm×0.4cm×0.3cm in size, located 6cm from the staple line and 0.4cm from the pleural capsule.

Note: The 0.1cm difference in maximum diameter between CT measurement (1.7cm×1.5cm×1.0cm) and gross measurement was clinically acceptable. This discrepancy was attributed to: ① Respiratory motion affecting in vivo CT measurement versus static measurement of the fixed gross specimen; ② No significant tissue shrinkage caused by 10% neutral formalin fixation, and the difference mainly resulted from minor misalignment in the selection of the maximum diameter plane. This discrepancy did not affect lesion staging.

- Bronchial stump (measuring 3.2cm×0.3cm×0.2cm): No tumor involvement was identified after staple removal.

#### 2.4.2 Microscopic and immunohistochemical findings

**Figure 2:**
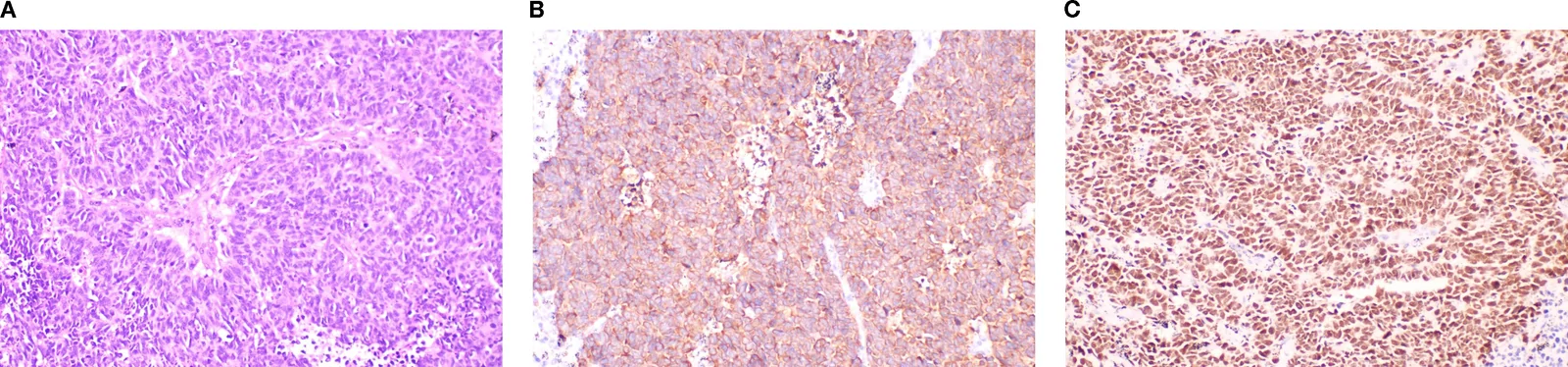
**(A)** Nodule 1 (SCLC): Hematoxylin-eosin (HE) staining showing nested tumor cells with high nuclear-cytoplasmic ratio (×200; scale bar = 100μm). **(B)** Nodule 1 (SCLC): Syn immunohistochemical staining (+, ×200; scale bar = 100μm). **(C)** Nodule 1 (SCLC): TTF-1 immunohistochemical staining (+, ×200; scale bar = 100μm).

**Figure 3:**
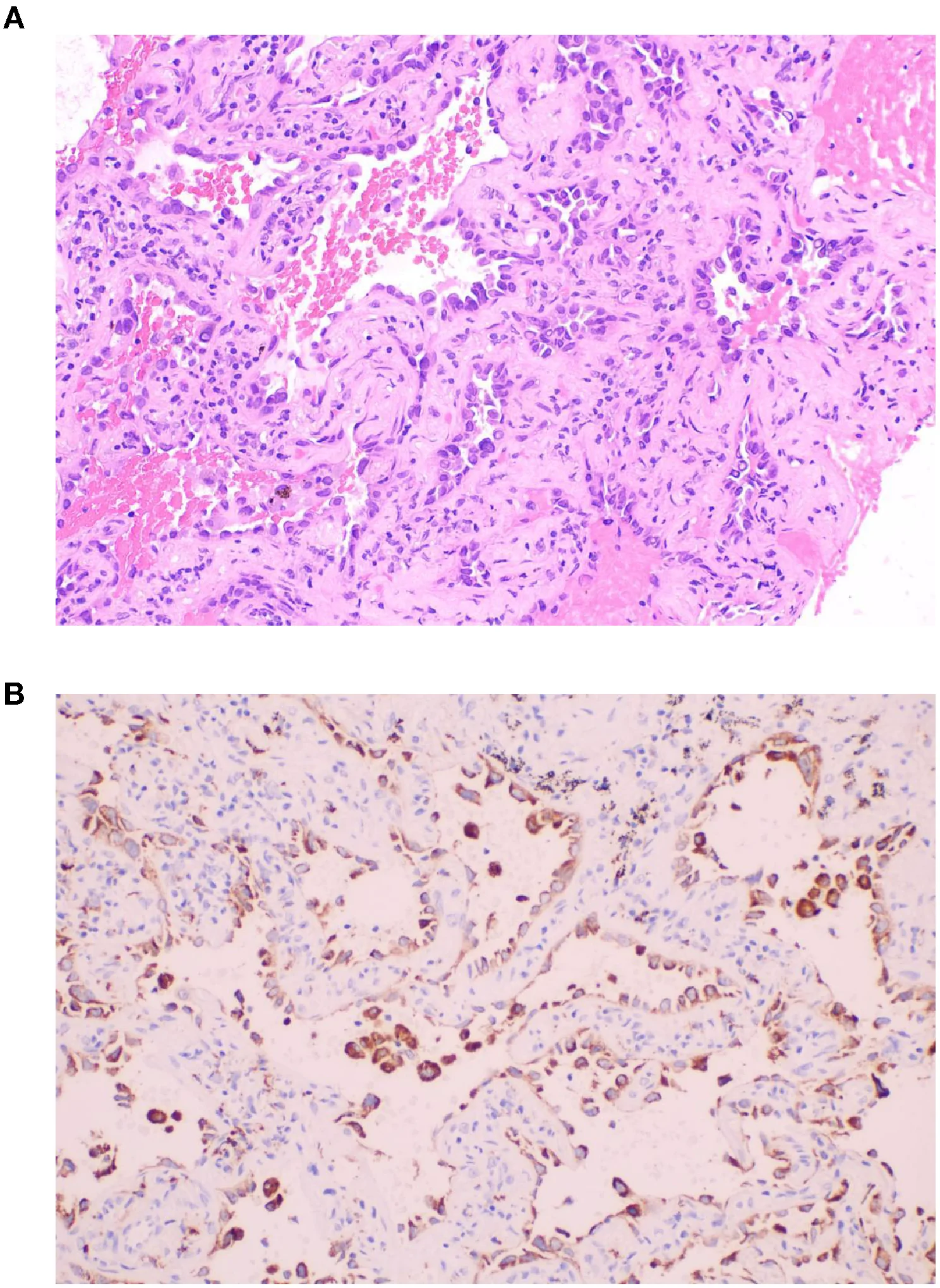
**(A)** Nodule 2 (adenocarcinoma): HE staining showing predominantly lepidic growth with focal acinar structures (×200; scale bar = 100 mm). **(B)** Nodule 2 (adenocarcinoma): Napsin A immunohistochemical staining (+, ×200; scale bar = 100 mm).

The pathological diagnosis was made in accordance with the 2015 WHO Classification of Lung Tumors (4), with updated classifications (22) (**Figures 2** and **3**). The diagnosis of MIA was confirmed after pathology department re-evaluation: the VG-positive area within the 0.8 cm nodule represents alveolar collapse with elastic fiber aggregation rather than true tumor desmoplasia, and the invasive focus (including the VG-positive region) does not exceed 5 mm. PD-L1 (22C3 IHC) testing was negative (TPS <1%, CPS <1). The clinicopathological characteristics of the two lesions are compared in detail in **Table 1**.

**Table 1:** Clinicopathological comparison of the two lesions.

Item	Nodule 1 (SCLC)	Nodule 2 (MIA)
Location	Posterior segment, RUL	Apical segment, RUL
Pathological type	Peripheral small cell lung cancer	Peripheral minimally invasive adenocarcinoma
Key features	Nested tumor cells with high N/C ratio, airway spread	Predominantly lepidic growth (70%), acinar growth (30%), no invasion beyond alveolar basement membrane
Syn	+	–
CgA	Focal +	–
CD56	+	–
TTF-1	+	+
Napsin A	–	+
EGFR	EGFR (+)	EGFR (NE)
ALK	–	–
Ki-67	~80%	~1%

### 2.5 Next-generation sequencing analysis

NGS analysis (a hybrid-capture-based 16-gene targeted panel covering exons and partial intronic regions of 16 lung cancer-related genes, detecting point mutations, insertions/deletions, fusions, and copy number variations) was performed on mixed paraffin-embedded tissue from both lesions (Nodule 1 and Nodule 2). The results showed: EGFR exon21 c.2573T>G mutation (p.L858R, 0.46%, OncoKB Level 1, sensitive to EGFR tyrosine kinase inhibitors such as osimertinib); TP53 exon8 c.820G>T mutation (p.V274F, 79.5%, a hallmark mutation characteristic of SCLC (12)); and ALK exon3 c.880C>G mutation (p.P294A, 69.5%) and exon11 c.1918G>A mutation (p.G640R, 11.9%, clinical significance unknown) (9). No variants were detected in MET, BRAF, or ERBB2 genes, importantly including no RB1 mutation, which is consistent with *de novo* SCLC but insufficient to exclude transformation (T-SCLC) from a pre-existing EGFR-mutant NSCLC clone (13). The markedly different allele frequencies (EGFR 0.46% vs. TP53 79.5%) strongly suggest that these mutations originate from different tumor components—TP53 predominantly from the SCLC component and EGFR from the MIA component—thereby providing molecular-level evidence supporting the diagnosis of dual primary lesions. PD-L1 (22C3 IHC) testing was negative (TPS <1%, CPS <1), and the tumor content of the mixed tissue sample was 85%. Detailed genomic findings of the mixed tissue sample are summarized in **Table 2**. **Table 2** summarizes the molecular profiling results.

**Table 2:** Summary of NGS findings.

Gene	Mutation	Exon	VAF	Clinical significance
EGFR	c.2573T>G (p.L858R)	21	0.46%	OncoKB Level 1 – sensitivity to EGFR-TKIs (osimertinib)
TP53	c.820G>T (p.V274F)	8	79.5%	Loss of tumor suppressor function – hallmark of SCLC [12]
RB1	Wild-type	—	—	Consistent with *de novo* SCLC, but insufficient to exclude transformation given analytical constraints [13]
ALK	c.880C>G / c.1918G>A	3 / 11	69.5% / 11.9%	Clinical significance unknown [9]
PD-L1 (22C3 IHC)	Negative			TPS <1%, CPS <1

NGS was performed on mixed paraffin-embedded tissue from both lesions. The striking VAF disparity supports independent clonal origins. RB1 wild-type status is consistent with *de novo* SCLC but insufficient to exclude transformation.

### 2.6 Postoperative treatment and recovery

- Adjuvant Chemotherapy: The etoposide plus cisplatin regimen was selected based on its proven efficacy (60% objective response rate for SCLC and 30%-40% sensitivity for adenocarcinoma) (7), and alignment with the NCCN Clinical Practice Guidelines (3, 8).
- Adverse Events: Grade II nausea and vomiting occurred, which were relieved with palonosetron and dexamethasone. No Grade IV myelosuppression or organ injury was observed.
- The patient was discharged on postoperative day 5 with an Eastern Cooperative Oncology Group Performance Status (ECOG PS) score of 1, without complications such as infection or pleural effusion. Two weeks after surgery, four cycles of adjuvant chemotherapy with etoposide (100mg/m², days 1–3) plus cisplatin (75mg/m², day 1) were administered every 21 days.

### 2.7 Follow-up

**Figure 4:**
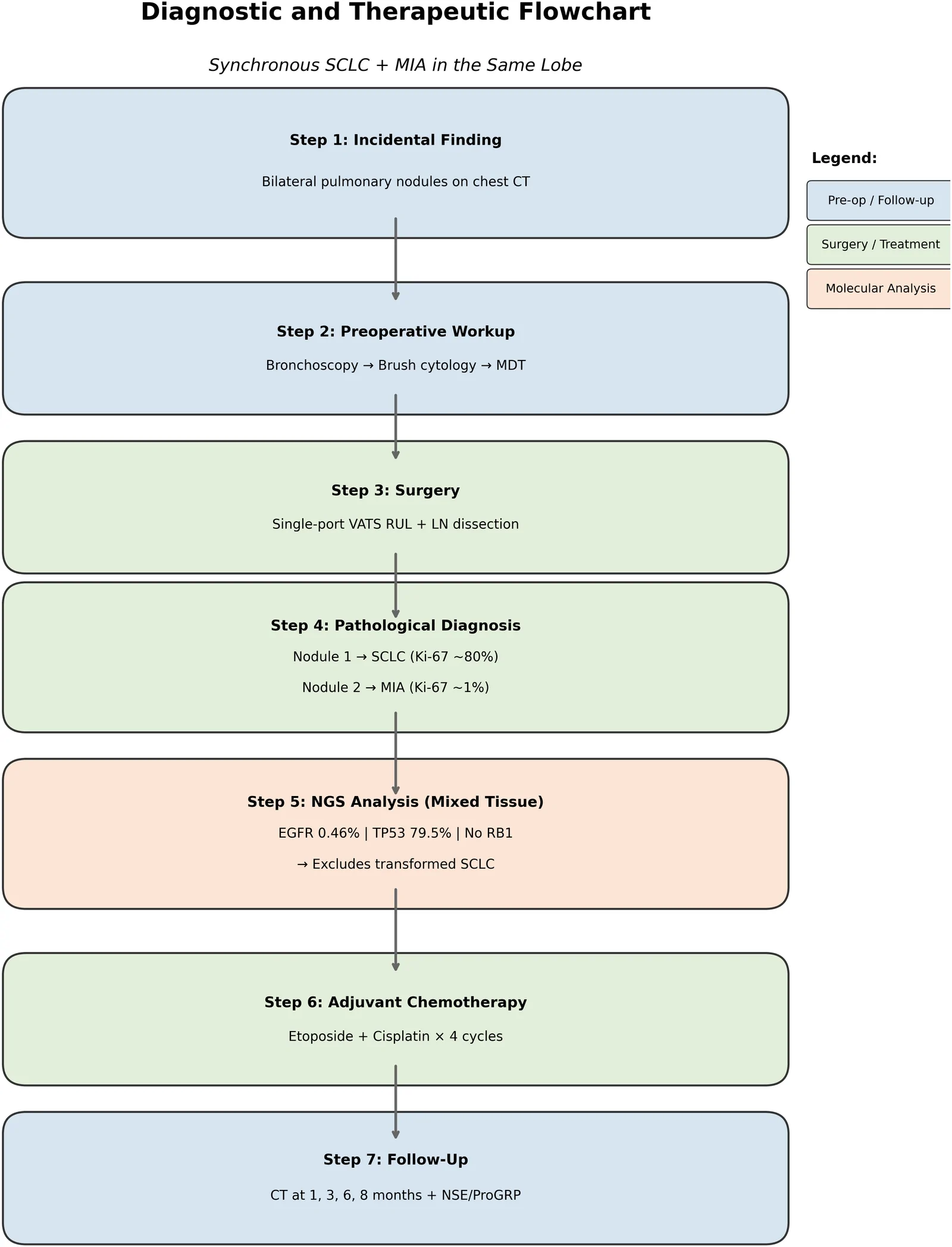
Diagnostic and therapeutic flowchart for the present case with clinical timeline. Key timepoints: January 2024 — initial CT detection of bilateral pulmonary nodules; February 2025 — CT showed progression of Nodule 1 (0.3→1.7cm, 31-fold volume increase) while Nodule 2 remained stable; February 28, 2025 — single-port VATS right upper lobectomy with lymph node dissection; March 2025 — pathological diagnosis of synchronous SCLC (Nodule 1) and MIA (Nodule 2), NGS analysis of mixed tissue; April–July 2025 — four cycles of adjuvant etoposide + cisplatin chemotherapy; August 2025 (3-month), October 2025 (6-month), and February 2026 (8-month) — regular CT and tumor marker follow-up.

Given the aggressive nature of SCLC, the patient underwent chest CT at 1, 3, 6, and 8 months postoperatively. At the most recent 8-month follow-up: Chest CT showed no tumor progression (assessed as stable disease per RECIST criteria); ECOG PS score remained 1 (**Figure 4**).

### 2.8 Patient perspective

The patient reported satisfaction with the treatment outcomes, noting minimal postoperative pain and rapid return to daily activities. He expressed appreciation for the multidisciplinary approach and clear communication regarding his diagnosis and treatment plan. At the 8-month follow-up, he reported good quality of life without significant limitations in daily functioning.

## 3 Discussion

Synchronous cross-histological small cell lung cancer (SCLC) and adenocarcinoma occurring in the same lobe is an extremely rare entity. To better understand this case, we first review the epidemiology of different lung cancer subtypes and the latest treatment landscape, before discussing diagnostic challenges and molecular evidence.

Lung cancer remains the leading cause of cancer-related death worldwide, with approximately 2.2 million new cases and 1.8 million deaths annually. Non-small cell lung cancer (NSCLC) accounts for approximately 85% of all lung cancers, while small cell lung cancer (SCLC) accounts for approximately 15% (14). With continued advances in research, clinicians have gradually changed the treatment landscape and strategies for these two subtypes. For advanced NSCLC, immune checkpoint inhibitors—whether used as monotherapy or in combination with chemotherapy—have demonstrated excellent efficacy across multiple clinical trials and significantly improved long-term survival (15, 21, 24). At the same time, for patients with actionable driver mutations (EGFR, ALK, ROS1, etc.), targeted therapy has increasingly become a precision treatment option for NSCLC (23). However, for SCLC patients, although the initial treatment is sensitive to chemotherapy, the prognosis remains poor with rapid recurrence, and the 5-year survival rate for extensive-stage SCLC is only approximately 7%. The addition of immunotherapy (e.g., atezolizumab or durvalumab) to platinum-based chemotherapy has extended overall survival in the first-line treatment of SCLC patients, representing the first major therapeutic breakthrough in this field in decades (16,25).

### 3.1 Diagnostic challenges and ‘benign mimicry’

Early-stage SCLC may initially present with ‘benign mimicry’ features on CT (e.g., a 0.3cm solid nodule with clear borders) (10), a finding that can easily be misinterpreted by clinicians as a low-risk nodule. However, its high proliferative activity (Ki-67 index of approximately 80%) drives rapid tumor progression (31-fold volume increase within 1 year), consistent with the median doubling time of SCLC (52 days) (11). Immunohistochemical examination and the Martini-Melamed criteria remain essential for confirming dual primary lesions (1). Therefore, for small solid nodules in high-risk populations, especially non-smoking patients with cardiovascular comorbidities, reliance on traditional follow-up strategies alone is insufficient; individualized and precise follow-up plans are needed.

### 3.2 Therapeutic rationale and outcomes

Single-port video-assisted thoracoscopic surgery (VATS), compared with traditional thoracotomy, achieves minimally invasive radical resection with excellent recovery (6). For elderly patients with comorbidities, this surgical approach offers clear advantages in reducing postoperative pain and shortening hospital stay. Reviewing this patient’s course, 4 cycles of adjuvant chemotherapy (etoposide plus cisplatin) were well tolerated (only Grade II nausea and vomiting), and stable disease was achieved at the 8-month follow-up. NGS analysis revealed an EGFR p.L858R mutation, preserving the option of targeted therapy for potential adenocarcinoma recurrence (20).

### 3.3 Molecular evidence supporting dual primary origin and excluding transformed SCLC

A key differential diagnosis in this case is whether the SCLC component may have arisen from transformation of a pre-existing EGFR-mutant NSCLC clone (transformed SCLC, T-SCLC), a well-recognized resistance mechanism to EGFR-TKIs (13). However, we consider *de novo* SCLC to be more likely for the following reasons: (1) The patient had no prior exposure to EGFR-TKIs, the typical trigger for T-SCLC—this is the strongest argument against transformation. (2) Both lesions were detected synchronously at the time of initial diagnosis, which is inconsistent with T-SCLC, which typically emerges months to years after TKI therapy. (3) NGS detected no RB1 mutation. Although RB1 inactivation can occur through mechanisms not detectable by panel NGS (e.g., promoter methylation or allelic deletion), and approximately 20–30% of confirmed T-SCLC cases lack RB1 mutations in published series, the absence of RB1 combined with the above clinical features is consistent with *de novo* SCLC, but is insufficient to completely exclude transformation given the analytical limitations of mixed-tissue NGS. (4) The low VAF of EGFR (0.46%) suggests it originated from the MIA subpopulation rather than serving as a clonal founder mutation driving both components. Therefore, this patient most likely has *de novo* synchronous dual primary lung cancers rather than transformed SCLC.

The coexistence of two histologically distinct tumors is driven by interconnected microenvironmental and genetic mechanisms:

Independent Clonal Origin with Genetic Heterogeneity: The striking VAF disparity between TP53 (79.5%) and EGFR (0.46%) in mixed tumor tissue provides strong evidence that these tumor mutations originate from distinct clonal populations. VAF, defined as the proportion of sequencing reads carrying a specific variant, is increasingly recognized as a surrogate marker for tumor clonal architecture and heterogeneity (17, 18). The high VAF of TP53 (79.5%) is consistent with its origin from the dominant SCLC component, while the low VAF of EGFR (0.46%) suggests a minor MIA-derived subpopulation within the mixed tissue sample. Two ALK variants were also detected (c.880C>G, VAF 69.5%; c.1918G>A, VAF 11.9%). Since NGS was performed on mixed tissue from both lesions, these variants likely reflect subclonal populations from different tumor components. Neither variant involves the ALK tyrosine kinase domain, and their clinical significance remains uncertain. Notably, the high VAF of the ALK c.880C>G variant (69.5%), comparable to that of TP53, more likely reflects copy-number instability in the SCLC genome rather than a straightforward somatic driver. The distinctly different biological behaviors of the two lung cancers—SCLC with Ki-67 ~80% and a 31-fold volume increase within 1 year, versus MIA with Ki-67 ~1% and stable size—further underscore the profound molecular divergence despite spatial proximity. This finding suggests that tumor-intrinsic genetic drivers, rather than shared microenvironmental factors, may be the primary determinants of clinical aggressiveness. According to a systematic review by Jiang et al. (9), the pooled mean volume doubling time (VDT) for SCLC is approximately 73 days, compared to 223 days for adenocarcinoma—a nearly 3-fold difference that highlights the urgency of timely monitoring for early-stage SCLC.

Non-Smoking-Related Carcinogenesis and Biological Mechanisms: Unlike most smoking-related sMPLC cases (2), the patient in this report had no smoking history. Although SCLC is classically associated with heavy tobacco exposure, growing evidence indicates that SCLC can also arise in never-smokers through alternative carcinogenic pathways, including air pollution exposure, occupational hazards, and chronic inflammation (19). In this patient, chronic systemic inflammation related to coronary heart disease may have contributed to the formation of a pro-carcinogenic pulmonary immune microenvironment. The simultaneous occurrence of SCLC (with typical TP53 inactivation) and EGFR-mutant MIA in a never-smoker raises an intriguing hypothesis: distinct carcinogenic pathways—one TP53-driven and one EGFR-driven—may be simultaneously activated in different progenitor cells within a similar microenvironment of the same lung lobe. This observation expands the etiological spectrum of sMPLC and suggests that non-smoking status should not lower clinical suspicion for SCLC when imaging findings are equivocal.

### 3.4 Clinical implications: optimizing the follow-up strategy for small peripheral solid nodules

The ‘benign mimicry’ of early-stage SCLC poses a significant diagnostic challenge. According to a comprehensive review by Megyesfalvi et al. (10), SCLC is characterized by rapid growth, high metastatic potential, and distinctive molecular features including universal TP53 and RB1 alterations, with median survival for extensive-stage SCLC remaining poor (8–13 months). This case reveals a critical pitfall: a small peripheral nodule with benign imaging features may in fact be an occult invasive SCLC with rapid proliferative capacity. Based on these considerations and existing evidence (10, 11), we identify a key gap in current follow-up guidelines: small (<0.5cm) peripheral solid nodules meeting ‘low-risk’ imaging criteria may harbor occult SCLC with explosive growth potential. In such cases, adherence to standard 6–12 month follow-up intervals may lead to delayed diagnosis of a rapidly fatal cancer. To address this, we propose the following optimized follow-up strategy for <0.5cm peripheral solid nodules:

This optimized strategy can specifically address the problem of delayed diagnosis of early-stage SCLC, which represents the core clinical value of this case in conjunction with existing studies (10, 11).

High-risk Scenario 1 — Guideline-Concordant Follow-Up May Be Insufficient: Nodules with benign imaging features but located in the upper lobe (a high-incidence area of sMPLC (1)). In this case, the 0.3cm nodule met the ‘low-risk’ criteria of current guidelines (clear borders, no lobulation), which would conventionally warrant 6–12 month follow-up. However, its underlying SCLC pathology (Ki-67 ~80%) drove a 31-fold volume increase within 1 year, meaning that adherence to standard follow-up intervals would have led to a critical delay in diagnosis. Therefore, for such nodules, even when imaging features appear ‘low-risk’, a 3-month follow-up is recommended.

High-risk Scenario 2 — Non-Smokers and Patients with Comorbidities: A 3-month follow-up is recommended. Combined with the rapid proliferative nature of SCLC (11), this can reduce the risk of missed diagnosis of occult lesions.

Dynamic Monitoring of Tumor Markers: For regular follow-up of patients with high-risk small nodules, synchronous detection of progastrin-releasing peptide (ProGRP) and neuron-specific enolase (NSE) (SCLC-specific markers) is recommended, along with imaging examinations to monitor nodule changes and improve the accuracy of early diagnosis (10).

This optimized strategy can specifically address the problem of delayed diagnosis of early-stage SCLC, which is the core clinical value of this case combined with existing studies (10, 11).

In conclusion, synchronous cross-histological SCLC and adenocarcinoma in the same lobe is an extremely rare entity, presenting unique diagnostic significance and treatment challenges. In conjunction with existing evidence (1, 4, 10), preoperative multimodal evaluation, postoperative immunohistochemical examination, and NGS are crucial for accurate diagnosis (5, 9). Single-port VATS combined with platinum-based chemotherapy is safer and more effective for elderly patients with comorbidities (6–8). For patients with high-risk peripheral solid nodules <0.5cm on imaging, a 3-month follow-up is recommended to avoid missed diagnosis of occult SCLC (10, 11). This case, together with existing literature, explores the pathogenesis of multiple primary lung cancers and further deepens the clinical understanding of sMPLC while providing a multi-dimensional supplement to current guidelines.

## 4 Limitations

This case report has several limitations. First, as a single case report, the findings may not be generalizable to the broader population of patients with synchronous multiple primary lung cancer. Second, NGS was performed on mixed paraffin-embedded tissue from both lesions rather than on individually sequenced specimens from each nodule. Although the striking difference in variant allele frequencies (EGFR 0.46% vs. TP53 79.5%) supports their independent origins, separate sequencing of each lesion would provide more definitive evidence of clonal origin. Third, the follow-up period of only 8 months is relatively short, and longer follow-up is needed to assess long-term outcomes and recurrence patterns. Fourth, PD-L1 expression was low in the adenocarcinoma component; however, separate PD-L1 testing of the SCLC component was not performed. Fifth, as a retrospective observational report, inherent selection bias cannot be excluded. Despite these limitations, this case has notable strengths: (1) it is one of the few reported cases of same-lobe synchronous SCLC and MIA with comprehensive NGS validation, providing molecular-level evidence of cross-histological origin; (2) the detailed analysis of VAF disparity offers a practical surrogate for clonal origin assessment when microdissection is unavailable; (3) the documentation of ‘benign mimicry’ and ultra-fast progression in early-stage SCLC supplements the existing literature and provides clinically valuable evidence for optimizing follow-up strategies.

## Data Availability

The original contributions presented in the study are included in the article/supplementary material. Further inquiries can be directed to the corresponding author.
